# Detecting Small Vessel Pathology in Cocaine Use Disorder

**DOI:** 10.3389/fnins.2021.827329

**Published:** 2022-02-10

**Authors:** Marco Öchsner, Elijah Mak, Karen D. Ersche

**Affiliations:** ^1^Department of Psychiatry, University of Cambridge, Cambridge, United Kingdom; ^2^Department of Systems Neuroscience, University Hospital Hamburg-Eppendorf, Hamburg, Germany

**Keywords:** cocaine, addiction, neuroimaging, FLAIR (fluid attenuated inversion recovery), hyperintensities detection, microbleeds, risk factors, impulsivity

## Abstract

**Background:**

Cocaine use is associated with an increased risk of cerebrovascular accidents. Small vessel pathology has been linked to the risk of stroke in cocaine users, but can be challenging to detect on conventional magnetic resonance (MR) scans. Fluid-attenuated inversion recovery (FLAIR) scans permit better resolution of small vessel lesions.

**Objectives:**

FLAIR scans are currently only acquired based on the subjective judgement of abnormalities on MR scans at face value. We sought to evaluate this practice and the added value of FLAIR scans for patients with cocaine use disorder (CUD), by comparing microbleeds detected by MR and FLAIR scans. We hypothesised that microbleeds are more pronounced in CUD patients, particularly so in participants who had been selected for a FLAIR scan by radiographers.

**Methods:**

Sixty-four patients with CUD and 60 control participants underwent a brain scan. The MR of 20 CUD patients and 16 control participants showed indicators of cerebral infarction at face value and were followed up by a FLAIR scan. We determined the volume of microbleeds in both MR and FLAIR scans and examined associations with various risk factors.

**Results:**

While MR lesion volumes were significantly increased in CUD patients, no significant differences in lesion volume were found in the subgroup of individuals who received a FLAIR.

**Conclusion:**

The current practice of subjectively evaluating MR scans as a basis for the follow-up FLAIR scans to detect vascular pathology may miss vulnerable individuals. Hence, FLAIR scans should be included as a routine part of research studies.

## Introduction

Cocaine is one of the most frequently used recreational drugs worldwide, with an estimated 20 million individuals having used cocaine in 2019 [United Nations Office on Drugs and Crime (UNODC), 2021]. Regular cocaine use is associated with serious morbidity and mortality (Jalal et al., 2018), particularly with an increased risk of cerebrovascular accidents (Klonoff et al., 1989; Treadwell and Robinson, 2007). For example, cocaine has been linked with a 5.7 times higher risk of ischaemic stroke (Cheng et al., 2016) and a 2.3 times higher risk of haemorrhagic stroke (Westover et al., 2007). Less is known about the relationship between regular cocaine use and cerebral small vessel pathology. Small vessel pathology in the brain is visible as microbleeds on magnetic resonance (MR) scans. Clinical evidence suggests that these may enhance the cocaine-related risk of stroke (Shoamanesh et al., 2016), which may serve as a useful marker to identify at-risk individuals early. However, microbleeds have received surprisingly little attention in both research and clinical practice. Importantly, not only cocaine use but other factors such as high blood pressure (Lyu et al., 2020) or high body mass index (BMI) (Kim et al., 2012) are thought to render individuals vulnerable to developing microbleeds. Given that cerebral microbleeds and stroke are typically accompanied by behavioural and cognitive changes (van Norden et al., 2011; Akoudad et al., 2013; Zivadinov et al., 2016), it is tempting to speculate whether an increased vulnerability for microbleeds is associated with increased disinhibition and impulsive personality traits. Impulsivity has also been considered to be both a determinant and consequence of cocaine addiction (American Psychiatric Association, 2000; de Wit, 2009) and may thus increase the risk of developing cocaine addiction in individuals who are using cocaine despite being vulnerable for microbleeds.

One likely reason why cerebral small-vessel pathology has received relatively little attention in research studies may be due to the difficulty in detecting and measuring them. Tissue damage due to hypoperfusion or infarction is detectable on conventional T1- and T2-weighted MR scans. However, these scans are less suitable for the detection of lesions in fluid filled spaces, such as ventricles or sulci, due to the similarity in the signal acquired for lesions and the cerebrospinal fluid on these scans (Pantoni, 2010). Fluid attenuated inversion recovery (FLAIR) scans overcome this limitation by suppressing the cerebrospinal fluid signal, and hence are particularly sensitive to small vessel disease in the brain (De Coene et al., 1992). Small-vessel pathology is reflected predominantly in cerebral microbleeds, focal perivascular hemosiderin depositions (Shoamanesh et al., 2011), with estimates suggesting a prevalence of about 5–6% in middle-aged individuals (de Leeuw et al., 2001; Poels et al., 2010), and white matter hyperintensities of vascular origin, which also have increasing prevalence with age (Garnier-Crussard et al., 2020), subsequently referred to as microbleeds. Unfortunately, FLAIR scans are not routine practice in clinical and research settings as they are only employed upon suspicion of small vessel infarcts or white matter lesions. Individuals that could be at risk of small vessel disease, and at increased risk of future cerebrovascular accidents, therefore remain undetected. As a consequence, only a very small number of cases have been reported where white matter lesions have been detected in regular cocaine users (González-Duarte and Williams, 2013; Vosoughi and Schmidt, 2015).

Here we sought to investigate whether FLAIR scans are more sensitive and reliable in assessing the prevalence of microbleeds in study participants both with and without cocaine use disorder (CUD). In light of the current practice, where independent radiographers evaluate MR scans at face value to judge whether or not an additional FLAIR scan should be acquired, the present study addressed the following aims: (i) to determine the characteristics of individuals with increased microbleeds, as detected in MR scans with respect to demographic variables, cardiovascular risk factors, impulsive personality traits, and indicators of cocaine use, (ii) to evaluate the added value of FLAIR scans for the detection of microbleeds, and (iii) to verify radiographers’ professional judgements for additional FLAIR scans. We hypothesised that CUD patients have more microbleeds when compared with control participants. We predict that lesion volume is particularly associated with cocaine-related indicators rather than with typical cardiovascular risk factors, and that this association would be stronger when considering FLAIR scans.

## Materials and Methods

### Study Sample

The study received ethical approval from the National Research Ethics Committee and all participants gave written informed consent before enrolment in the study. We recruited 64 individuals with a history of chronic cocaine use from local drug treatment services, who met the DSM-IV-TR criteria for cocaine dependence (American Psychiatric Association, 2000), subsequently referred to as cocaine use disorder (CUD). We also recruited 60 control volunteers from the community, who had no personal history of regular drug use. Exclusion criteria for all study participants were a lifetime history of major neurological illness, traumatic head injury, or psychotic disorder, and no contraindications to MRI scanning.

All participants followed the same study protocol, as described elsewhere (Ersche et al., 2013, 2014, 2015, 2017). All participants were between 20 and 59 years, and predominantly male (97%) in keeping with the majority of cocaine users being male [United Nations Office on Drugs and Crime (UNODC), 2021]. Patients with CUD had been using cocaine on average for 17 years (±7.4 years) and started at the age of 21 years (±5.4 years). They were actively using cocaine, which was confirmed by a urine sample on the day of the MRI scan. Most CUD patients were also active tobacco smokers (92%), and met the diagnostic criteria for dependence on other substances such as opiates (58%), cannabis (32%), and alcohol (17%). CUD patients were asked to complete the Obsessive Compulsive Drug Use Scale (OCDUS) questionnaire, which quantifies compulsive cocaine use patterns (Franken et al., 2002). Control participants were mostly non-smokers (90.3%) and scored very low on the Drug Abuse Screening Test (DAST-20, Skinner, 1982) and the Alcohol Use Disorder Test (AUDIT, Saunders et al., 1993); all urine samples provided by control participants prior to scanning tested negative for drugs. All participants were screened psychiatrically prior to scanning using the Mini International Neuropsychiatric Interview (MINI, Sheehan et al., 1998) and information about acute cerebrovascular risk markers (e.g., BMI, blood pressure) were collected. Participants were also asked to complete the Barratt Impulsiveness Scale version 11 (BIS-11, Patton et al., 1995) as a measure of trait impulsivity.

### Neuroimaging Data Acquisition

The MR scans were acquired at the Wolfson Brain Imaging Centre, at the University of Cambridge, United Kingdom using a Siemens Magentom Trio Tim scanner at 3 Tesla. For T1-weighted MRs, a magnetically prepared rapid acquisition gradient echo sequence (MPRAGE) was used (176 slices of 1 mm thickness, repetition time = 2300 ms, echo time = 2.98 ms, inversion time = 900 ms, flip angle = 9 degrees, field of view = 240 × 256). These MR scans were then assessed by an independent radiographer, and if any indication of cerebral microbleeds was present the participant received a FLAIR scan.

### Statistical Analysis

The data were analysed using a five-step strategy, as outlined below. All statistical tests were two-tailed with a significance threshold of *p* < 0.05, and performed using the SciPy library in Python version 3.2 (Virtanen et al., 2020).

1. To assess group differences in demographics, a two-tailed independent sample *t*-test was used, as the sample data were normally distributed. Chi square and Fisher’s exact tests were used to compare categorical variables, as appropriate. We first compared the entire study sample, before comparing CUD patients and healthy controls who received a FLAIR. We also assessed for bias in participant selection for a FLAIR by comparing participants who received a FLAIR with those who did not, as well as the proportions of control participants and CUD patients from the whole sample who received a FLAIR.

2. T1-weighted MRI hypointensity volumes were generated using the FreeSurfer image analysis suite (Fischl et al., 2004) to obtain T1-MRI microbleed-lesion volumes. Volumes were then normalised to the total brain volume and imported into Python for group comparison and correlational analysis.

3. Fluid attenuated inversion recovery lesion volumes were obtained by segmentation of hyperintense lesions using the Lesion Growth Algorithm in the Lesion Segmentation Toolbox (Schmidt et al., 2012) in the Statistical Parametric Mapping (Friston et al., 2007) software package version 12, in MatLab R2020b, to obtain FLAIR microbleed-lesion volumes. Segmentation thresholds were set at *k* = 0.1, 0.2, and 0.3 to generate lesion probability maps, and the best fitting lesions maps were chosen based on visual inspection, and then manually corrected to improve lesion mapping accuracy. Normalised lesion volumes were then imported into Python for group comparison and correlational analysis.

4. To compare lesion volumes between CUD patients and control participants, an inverse rank-normalisation was applied to both T1-MRI and FLAIR lesion volumes using the function RankNorm from the R library RNOmni (McCaw et al., 2020), as these are not normally distributed. Differences in lesion volumes between CUD patients and control participants were then assessed using a two-tailed independent sample *t*-test, with a significance threshold of *p* < 0.05.

5. Correlation analyses between lesion volumes and risk factors for microbleeds were conducted using a Pearson correlation, to investigate relationships between microbleeds and cocaine-related indicators [e.g., duration of regular cocaine use, age of cocaine use onset, compulsive cocaine use (OCDUS score)], vascular risk factors (e.g., blood pressure, BMI) and self-reported impulsivity (BIS-11 total score).

## Results

### Demographics, Personality, and Cocaine Use

Descriptive statistics for demographics, personality traits, and clinical data for all participants are shown in Table 1. The two groups were well-matched with respect to age, gender, and blood pressure. As expected, CUD patients scored significantly higher on trait impulsivity (*t*_122_ = −10.87, *p* < 0.001) and were more likely to smoke tobacco (91.7%) compared with healthy control participants (9.8%). With regards to risk factors for vascular pathology, measures of blood pressure and pulse were in both groups within the normal range but CUD patients had a significantly lower BMI compared with their non-drug using peers [control mean: 25.0 ± 3.2 standard deviation (±SD), CUD mean: 23.5 ± 3.5 SD; *t*_122_ = 2.34, *p* = 0.021].

**TABLE 1:** Demographics, personality traits, and clinical data [means and standard deviation (Std.) in parentheses] of the full sample and the subgroups that received a FLAIR scan.

Demographics	Group comparison (full sample)				Group comparison (all participants with FLAIR scan)			
	Control participants mean (±SD)	CUD patients mean (±SD)	*t* statistic	*p*-value	Control participants mean (±SD)	CUD patients mean (±SD)	*t* statistic	*p*-value
Sample size (*n*)	60	64	-	-	16	20	-	-
Age (years)	40.4 (±10.9)	37.9 (±8.4)	1.43	0.157	41.1 (±11.8)	39.8 (±8.1)	0.38	0.706
Gender (% male)	95.0%	96.9%	Fisher’s	0.672	93.8%	100%	Fisher’s *p*	0.444
FLAIR scan (% recipients)	26.7%	31.3%	Fisher’s	0.693	-	-	-	-
Formal education (years)	14.1 (±3.2)	11.3 (±1.8)	5.96	<0.001	13.3 (±2.9)	11.4 (±2.2)	2.22	0.035
Body Mass Index (BMI)	25.0 (±3.2)	23.5 (±3.5)	2.34	0.021	25.6 (±3.9)	24.2 (±2.8)	1.23	0.230
Systolic blood pressure (mmHg)	130.5 (±14.3)	126.7 (±14.5)	1.48	0.141	128.3 (±15.7)	128.5 (±14.2)	−0.02	0.988
Smoking status (% smokers)	6.7%	92.2%	Fisher’s	<0.001	12.5%	100%	Fisher’s *p*	<0.001
Impulsivity (BIS-11 total score)	57.9 (±8.2)	76.2 (±10.5)	−10.87	<0.001	59.4 (±8.3)	80.1 (±8.7)	−7.23	<0.001

Participants who received a FLAIR did not significantly differ on any demographic measures from those who did not receive a FLAIR (see Supplementary Table 1). FLAIRs were acquired for a similar proportion of control participants and CUD patients [26.7% of control participants and 31.3% of CUD patients; χ^2^ (2, *N* = 124) = 0.3157, *p* = 0.574]. However, CUD patients who received a FLAIR reported increased levels of impulsivity (CUD without FLAIR, mean BIS-11 total score: 74.4 ± 10.8 SD; CUD with FLAIR, mean BIS-11 total score: 80.1 ± 8.7 SD; *t*_62_ = −2.21, *p* = 0.032), and had used cocaine significantly longer compared with CUD patients without a FLAIR (CUD without FLAIR mean: 15.1 years ± 5.9 SD; CUD with FLAIR mean: 19.6 years ± 7.5 SD; *t*_58_ = −2.38, *p* = 0.024).

### Microbleeds in Cocaine Use Disorder Patients and Control Participants

**FIGURE 1:**
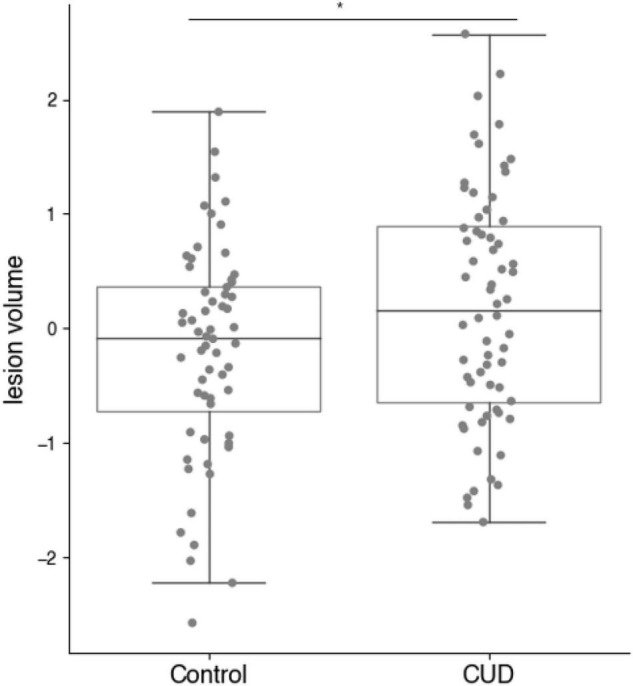
MR derived lesion volumes (normalised) in CUD patients and healthy control participants (full sample *n* = 124). Lesion volumes were significantly increased in the CUD patients compared with age-and sex-matched control participants (*p < 0.05).

**FIGURE 2:**
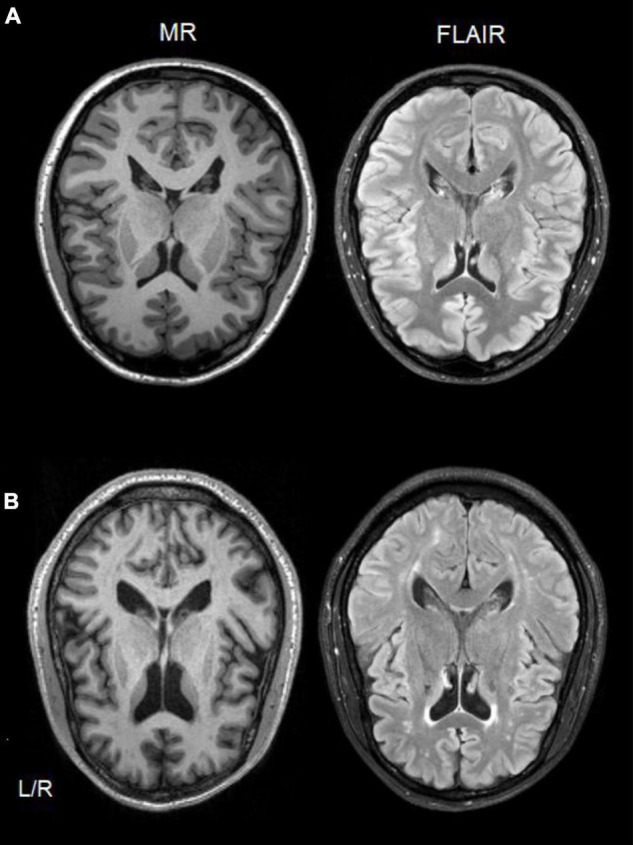
Single-subject comparison of T1-weighted MR (left side) and FLAIR (right side) scans from a healthy control participant **(A)**, and a CUD patient **(B)** showing white matter hyperintensities in both scan types.

**FIGURE 3:**
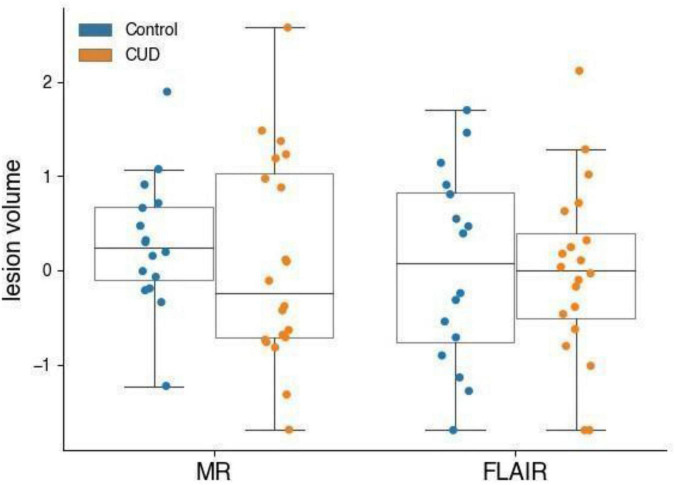
MR and FLAIR lesion volumes (normalised) of participants who received a FLAIR scan. CUD patients (*n* = 20) and control participants (*n* = 16) with microbleeds who received a FLAIR do not have measurably different microbleed lesion volumes.

As shown in Figure 1, MR lesion volumes in all CUD patients were significantly larger compared with lesion volumes in all control participants (*t*_122_ = −2.20, *p* = 0.030). MR lesion volumes were only associated with the total white matter volume in control participants (*r* = 0.30, *p* = 0.019), but not in CUD patients (*r* = 0.10, *p* = 0.414), suggesting that the white matter volume did not bias the comparison between CUD patients and controls. When we restricted the comparisons to those participants, who according to independent radiographers showed suspicious abnormalities and were followed up with a FLAIR scan (see Figure 2), the significant group differences disappeared (see also Figure 3), i.e., neither the MR scans (*t*_34_ = 0.69, *p* = 0.496) nor the FLAIR scans (*t*_34_ = 0.16, *p* = 0.871) revealed any significant differences between the two groups, although both scans were highly correlated (*r* = 0.5, *p* = 0.002). It is also of note that MR lesion volumes were significantly increased in control participants with a FLAIR scan when compared with those without a FLAIR scan (*t*_58_ = −2.94, *p* = 0.006), but this difference in lesion volume was not evident in CUD patients with and without FLAIR scans (*t*_62_ = 0.53, *p* = 0.598).

### Relationship Between Lesion Volumes, Personality, Cocaine Use, and Cardiovascular Risk Factors

Self-reported impulsivity levels (BIS-11) did not correlate with MR lesion volume in the entire sample but we observed a significant relationship in those control participants who received a FLAIR (*r* = 0.53, *p* = 0.036; controls without FLAIR: *r* = 0.33, *p* = 0.206). Impulsivity was not related to lesion volume in CUD patients (all *p* > 0.1).

With respect to cardiovascular risk factors, we only observed a significant relationship between MR lesion volumes and body mass index in CUD patients who received a FLAIR (*r* = 0.46, *p* = 0.041). However, this relationship was not evident when considering the FLAIR scans of CUD patients (*r* = 0.15, *p* = 0.533), and it was not seen not in control participants (MR controls: *r* = 0.13, *p* = 0.637; FLAIR controls: *r* = 0.13, *p* = 0.624). There was no a relationship between the number of cigarettes CUD patients smoked per day, and lesion volumes measured either by MR (*r* = −0.05, *p* = 0.700) or FLAIR scans (*r* = −0.34, *p* = 0.148), suggesting that the high levels of tobacco use in the CUD group did not confound the results. Alcohol consumption, as reflected by the AUDIT total score was not related to lesion volumes in either group (all *p* > 1).

The duration of cocaine use was significantly associated with lesion volume in MR scans of all CUD patients (*r* = 0.30, *p* = 0.017), but not the age of cocaine use onset or compulsive cocaine use. However, when we restricted the correlations to those CUD patients who received a FLAIR scan, this correlation did not survive.

## Discussion

Cerebral small vessel pathology is a significant risk factor for severe neurological conditions, but as they may be present without symptoms in vulnerable patients, their detection remains challenging. We found that, based on MR scans, the volume of these lesions was significantly increased in CUD patients when compared with age-matched healthy control participants. While the volume of these lesions was associated with the duration of cocaine use in CUD patients in MR scans, this association was not reflected in FLAIR scans. As participants were selected for FLAIR scan based on radiographers’ judgement of MR scans, these findings suggest that such subjective assessments are inadequate to identify at-risk individuals early and that FLAIR scans should be routinely acquired in vulnerable populations.

### Microbleed Risk Factors and Magnetic Resonance Lesion Volume

Although MR scans can detect small vessel disease as microbleeds, it remains unclear what factors predispose individuals most significantly to developing such lesions. As hypothesised, MR lesion volumes were indeed increased in CUD patients and associated with the duration of cocaine use, in keeping with prior work reporting increased lesions in CUD patients (Bartzokis et al., 1999; Vosoughi and Schmidt, 2015; Grasso et al., 2020). However, we did not find an association between lesion volumes and cardiovascular risk factors in our study, which might seem surprising given that microbleeds and stroke have both been widely linked to hypertension and obesity (Wartolowska and Webb, 2021). This may be due to our participants having lower mean values of blood pressure and BMI when compared with other studies on microbleeds. It is also conceivable that blood pressure in CUD patients may only temporarily be raised due to the acute effects of the drug, but not persistently, which may be why their blood pressure levels were normal at the time of the assessment. It is also of note that previous studies on microbleeds often recruited older participants with higher baseline measures of cardiovascular health, or focused on populations with vascular disease (Frey et al., 2019; Moroni et al., 2020). However, we cannot rule out that other cardiovascular risk factors such as 24 h systolic blood pressure (Abraham et al., 2016), levels of fasting glucose (Park et al., 2007) or blood lipids (Jimenez-Conde et al., 2010), which were not assessed in the present study, might have influenced lesion volume.

The white matter hyperintensities on MR scans are thought to reflect injury to myelinated axons and glial scarring (van Walderveen et al., 1998), but from the scan, it is not possible to identify the underlying cause. Whilst in multiple sclerosis, hyperintensities are caused by autoimmune processes (Datta et al., 2017), in dementia and age-associated cognitive decline these are often due to vascular pathology such as arteriosclerosis (Barker et al., 2014). MR lesions in CUD patients most likely reflect ischaemic damage to the white matter, as chronic cocaine use has been shown to lead to a persistent cerebral hypoperfusion associated with decreases in cognitive function (Strickland et al., 1993). Some case studies even suggest that cocaine use may cause vascular inflammation in the brain (Younger, 2019). White matter in the brain is less well perfused than the grey matter, and therefore is more susceptible to injury (Wu et al., 2013). This may explain why the risk factors in the present study were uncorrelated with lesion volume. There were also no demographic variables that predicted hyperintensitis, which further highlights the need for objective markers.

### The Added Value of Fluid Attenuated Inversion Recovery Imaging for the Detection of Microbleeds

Fluid attenuated inversion recovery scans have been especially developed to suppress cerebrospinal fluid signals, which makes lesions more visible even when these are in close proximity to the ventricles (De Coene et al., 1992). In the present study, we did not find group differences in FLAIR lesion volumes, which is particularly surprising because lesion volume in both scans were correlated in our study as well as in others (Wei et al., 2019). We believe that this is not a reflection on the general sensitivity of FLAIR scans, but more likely a result of how participants were selected for FLAIR scans by radiographers. It is worth noting that lesions measured by FLAIR and MR scans are not necessarily perfect matches, as each scanning modality varies in sensitivity when visualising lesions in different brain regions. For example, in studies of multiple sclerosis, it has been noted that FLAIR scans have increased sensitivity to periventricular hyperintensities, while MR more reliably shows lesions in the posterior fossa (Klawiter, 2013). The location of lesions may also affect the functional consequences that arise, and hence affect the association of disease progression or risk factors with MR or FLAIR lesion volumes. This may be reflected in differing correlations with disease severity reported for patients with multiple sclerosis (Narayana et al., 2014; Akaishi et al., 2020). In other words, where T1-weighted MR lesions appear to represent more severe and persistent lesions in the context of multiple sclerosis, FLAIR scans may additionally capture lesions which resolve at a later point. More research is warranted to better understand how FLAIR and MR scans complement one another.

### Identification of At-Risk Participants by Radiographers

Due to the increased risk of stroke and other cerebrovascular injury associated with microbleeds, it is important to identify individuals with increased lesion loads. Based on our findings, we believe that radiographers’ selection of participants for FLAIR scanning were not sufficiently sensitive to detect participants with microbleeds. Subjective ratings and assessments of lesions on scans have previously been shown to differ from objective measures (Filippi et al., 1995), particularly in the absence of clear guidelines. Individual judgments, even those of professionals, may vary considerably (Di Leo, 2015). For patients with various neurological disorders, FLAIR and MR scans are both routinely acquired to prevent missing significant pathology less reliably detected by MR alone. This highlights why the current practice may be problematic, especially in light of the risk of missing early signs of cerebrovascular accidents in psychiatric patients. Considering also the difficulty in detecting lesions based on MR, we believe that FLAIR scans should be made compulsory in research studies with vulnerable populations.

### Strengths and Weaknesses

The strengths of this study include the relatively large community sample of individuals who are most at risk, an age- and sex-matched control group, and the collection of information about various risk factors. By focusing on participants from the community without acute neurological injuries, we were able to evaluate the prevalence of subclinical lesions in asymptomatic individuals, specifically in individuals with chronic cocaine use. Moreover, the quantification of lesion volumes allowed us to assess differences between groups more reliably and establish associations between lesion loads and risk indicators. This is much more sensitive than relying on subjective visual rating scales of lesion frequency or severity that are frequently used (van den Heuvel et al., 2006). Potential limitations of this study are the lack of FLAIR scans from all participants, which would have permitted a more extensive comparison between MR and FLAIR scans and verified radiographers’ assessment. As most chronic cocaine users are men, our sample is predominantly male. It would be good if future studies could investigate lesion volume specifically in cocaine using women. Furthermore, we could not determine the contribution of other drugs study participants used on the prevalence and volume of microbleeds, as most CUD patients met criteria for dependence of at least one other substance. However, our sample is representative of chronic cocaine users in the United Kingdom and highlights their increased risk. FLAIR scans would therefore be particularly needed to identify potential small vessel disease early.

## Conclusion and Outlook

While MR scans are sufficiently sensitive to detect small vessel disease, we believe that FLAIR scans have a number of benefits, and as they are easily and quickly obtained, they should become standard practice in vulnerable populations. FLAIR scans may also offer the opportunity for researchers to investigate neuropathology more thoroughly, which may help to develop standards to identify individuals at risk in a reliable manner to avoid subjective misjudgements.

## Data Availability Statement

The raw data supporting the conclusions of this article will be made available by the authors, will be made available on request, without undue reservation.

## Ethics Statement

The studies involving human participants were reviewed and approved by the National Research Ethics Committee NRES East of England – Norfolk. All patients/participants provided written informed consent to participate in this study.

## Author Contributions

KDE acquired funding, designed the study, and wrote the protocol. MÖ managed the literature searches, analysed the data with the help of EM, and wrote the first draft of the manuscript with the help of KDE. All authors contributed to the article and approved the final manuscript.

## Conflict of Interest

The authors declare that the research was conducted in the absence of any commercial or financial relationships that could be construed as a potential conflict of interest.

## Publisher’s Note

All claims expressed in this article are solely those of the authors and do not necessarily represent those of their affiliated organizations, or those of the publisher, the editors and the reviewers. Any product that may be evaluated in this article, or claim that may be made by its manufacturer, is not guaranteed or endorsed by the publisher.
