# Supplementary material for: Detecting Small Vessel Pathology in Cocaine Use Disorder
Source: Front Neurosci. 2022 Feb 10;15:827329. doi: 10.3389/fnins.2021.827329 (PMC8867820; doi:10.3389/fnins.2021.827329)
Supplement: Supplementary file 1 [file Table_1.pdf]

Supplemental Material

Detecting Small Vessel Pathology in Cocaine Use Disorder

Marco Öchsner, Elijah Mak, Karen D Ersche✉

✉ ke220@cam.ac.uk

Table S1: Demographics, personality traits and clinical data for participants who received a FLAIR, and those who did not [means and standard deviation, (Std.) in parentheses]

**Demographics**	**Radiographer's choices**			
	**no FLAIR** mean (±SD)	**with FLAIR** mean (±SD)	**t statistic**	***p*-value**
Sample(n)	88	36	-	-
Age (years)	38.61 (± 9.7)	40.37 (± 9.8)	-0.92	0.364
Gender (% male)	95.5%	97.2%	Fisher's	1.000
Formal education (years)	12.86 (± 3.0)	12.22 (± 2.7)	1.16	0.249
Body Mass Index (BMI)	23.99 (± 3.5)	24.83(± 3.4)	-1.25	0.214
Systolic blood pressure (mmHg)	128.61 (± 14.5)	128.42 (± 14.7)	0.07	0.946
Smoking status (% smokers)	46.6%	61.1%	Fisher's	0.168
Impulsivity (BIS11 total score)	65.89 (± 12.8)	70.89 (± 13.4)	-1.91	0.060
	**CUD no FLAIR** mean (±SD)	**CUD with FLAIR** mean (±SD)	**t statistic**	***p*-value**
Sample (n)	44	20	-	-
Duration of cocaine use (years)	15.1 (± 5.9)	19.6 (± 7.5)	-2.38	0.024
Age at onset (years)	22.0 (± 6.0)	20.2 (± 4.8)	1.28	0.208
Compulsivity of drug use (OCDUS total)	23.9 (± 7.8)	25.7 (± 11.2)	-0.64	0.528
